# Button Battery Ingestion

**DOI:** 10.4274/balkanmedj.2017.0523

**Published:** 2018-03-15

**Authors:** Satvinder Singh Bakshi

**Affiliations:** 1 Department of Otolaryngology-Head and Neck Surgery, Mahatma Gandhi Medical College and Research Institute, Pondicherry, India

A 9 year old child presented with history of button battery ingestion for 2 hours associated with odynophagia. The plain X-ray of the neck revealed the foreign body with a ‘double halo’ or ‘double contour’ in the upper oesophagus suggestive of a button battery (Figure 1), which was removed immediately by rigid oesophagoscopy in the operating theatre (Figure 2). Rigid oesophagoscopy also revealed surrounding erythema, oedema and slough at the site of impaction. The child recovered without any complications and a repeat flexible endoscopy performed at 6 weeks was normal. Informed consent was taken from the parents of the patient.

In 1977, the first case of a button battery foreign body in the oesophagus was reported. Although button batteries account for only a small percentage of cases, there has been a steady increase in incidence over the past two decades (1). This can be attributed to the increased usage of button batteries in household appliances. The primary mechanism by which the button batteries cause damage is by leakage of the battery contents into the moist oesophageal environment which causes direct corrosive damage (2). The leaked alkaline electrolyte solution can penetrate deep into tissues producing liquefying necrosis. This damage can occur within a very short period of time and, therefore, early identification is of utmost importance (2). Children generally present with non-specific signs like pain, vomiting, odynophagia, and drooling of saliva. An X-ray of the neck and abdomen showing the ‘double contour’ appearance is confirmatory and helps to localise the site of impaction and type of foreign body. Complications include oesophageal perforation, mediastinitis, trachea-oesophageal fistula and oesophageal stenosis (1,2). Early intervention in the form of oesophagoscopy and removal of the button battery is required to prevent these complications. The most effective management strategy to reduce morbidity in these patients would be education of the parents, health care providers and the public about the potential hazards associated with button battery exposure.

## Figures and Tables

**Figure 1:**
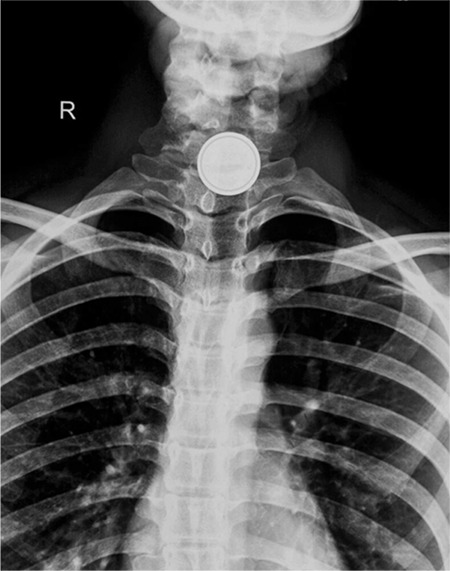
Plain X-ray of the neck. Anteroposterior view showing the ‘double contour’ suggestive of button battery in the oesophagus.

**Figure 2:**
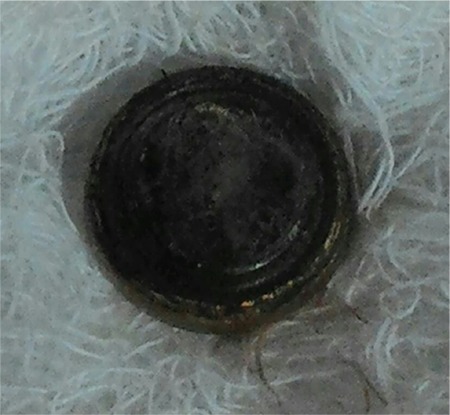
Picture of the leaked button battery.
